# Impact of atypical long-acting injectable versus oral antipsychotics on rehospitalization rates and emergency room visits among relapsed schizophrenia patients: a retrospective database analysis

**DOI:** 10.1186/1471-244X-13-221

**Published:** 2013-09-10

**Authors:** Marie-Hélène Lafeuille, François Laliberté-Auger, Patrick Lefebvre, Christian Frois, John Fastenau, Mei Sheng Duh

**Affiliations:** 1 Groupe d’analyse, Ltée, 1000 De La Gauchetière West, Suite 1200, Montréal, QC, Canada; 2 Analysis Group, Inc, 111 Huntington Avenue, Tenth Floor, Boston, MA, USA; 3 Janssen Scientific Affairs, LLC, 1125 Trenton-Harbourton Road, Titusville, NJ, USA

**Keywords:** Long-acting atypical antipsychotic, Oral antipsychotics, Schizophrenia, Hospitalization, Andersen-Gill extension, Cox proportional hazards models

## Abstract

**Background:**

Among schizophrenia patients relapsed on an oral antipsychotic (AP), this study compared the impact of switching to atypical AP long-acting injectable therapy (LAT) versus continuing oral APs on hospitalization and emergency room (ER) visit recurrence.

**Methods:**

Electronic records from the Premier Hospital Database (2006-2010) were analyzed. Adult patients receiving oral APs during a schizophrenia-related hospitalization were identified and, upon relapse (i.e., rehospitalization for schizophrenia), were stratified into (a) patients switching to atypical LAT and (b) patients continuing with oral APs. Atypical LAT relapse patients were matched 1:3 with oral AP relapse patients, using a propensity score model. Andersen-Gill Cox proportional hazards models assessed the impact of atypical LAT versus oral AP on time to multiple recurrences of all-cause hospitalizations and ER visits. No adjustment was made for multiplicity.

**Results:**

Atypical LAT (*N* = 1032) and oral AP (*N* = 2796) patients were matched and well-balanced with respect to demographic (mean age: 42.1 vs 42.4 years, *p* = .5622; gender: 43.6% vs 44.6% female, *p* = .5345), clinical, and hospital characteristics. Over a mean 30-month follow-up period, atypical LATs were associated with significantly lower mean number of rehospitalizations (1.25 vs 1.61, *p* < .0001) and ER visits (2.33 vs 2.67, *p* = .0158) compared with oral APs, as well as fewer days in hospital (mean days: 13.46 vs. 15.69, *p* = .0081). Rehospitalization (HR 0.81, 95% CI 0.76–0.87, *p* < .0001) and ER visit (HR 0.88, 95% CI 0.87–0.93, *p* < .0001) rates were significantly lower for patients receiving atypical LAT versus oral APs.

**Conclusions:**

This hospital database analysis found that in relapsed schizophrenia patients, atypical LATs were associated with lower rehospitalization and ER visit rates than oral APs.

## Background

Schizophrenia usually appears in early adulthood, and approximately two-thirds of individuals with this disease have persisting or fluctuating symptoms even with optimal treatment [1,2]. Inpatient care represents the primary driver of costs associated with schizophrenia, accounting for between one-third and two-thirds of the total direct health care costs of patients with schizophrenia [3-6]. Indeed, most of these patients will experience a chronic course with many relapses, characterized by an exacerbation of psychosis, emergency room (ER) visits, and rehospitalizations [7-9]. These relapse events are often associated with significant changes in the treatment of schizophrenia and initiation of new therapies [10,11].

The primary goal of pharmacotherapy with antipsychotics (APs) in patients with schizophrenia is to prevent relapse and to reduce the severity of subsequent acute episodes over time [12,13]. Compared with typical APs, atypical APs are generally considered to be associated with a lower risk of serious adverse events and are therefore the first-line therapeutic agents of choice for patients with schizophrenia in most countries [14-16]. In practice, the effectiveness of oral AP treatment is often undermined by poor adherence [17], which is associated with an increased frequency of relapse and hospitalization rates, more severe symptoms, longer inpatient stays, and higher hospital costs [18-21].

It has been shown that AP long-acting injectable therapy (LAT) can significantly improve adherence, reduce symptoms, and reduce the risk of relapse and rehospitalization, particularly for severely ill patients [9,22,23]. There are currently three atypical APs available in long-acting forms: risperidone (RISPERDAL® CONSTA®), paliperidone palmitate (INVEGA® SUSTENNA®), and olanzapine (ZYPREXA® RELPREVV®) [24-26]. Risperidone is indicated for the treatment of schizophrenia as one injection every 2 weeks, whereas paliperidone palmitate is a once-a-month agent approved for the acute and maintenance treatment of schizophrenia in adults [24,25]. Olanzapine is approved for the treatment of schizophrenia as one injection every 2 to 4 weeks but is available only through a restricted distribution program [26]. Because there is to date limited use of olanzapine LAT in clinical practice and given its markedly different profile from the two other atypical AP LATs, this analysis focused on risperidone and paliperidone palmitate LATs.

Most previous studies have found a beneficial effect of atypical LATs in terms of rehospitalizations using a pre-post study design, where each patient acted as his or her own control [9,20,22,27-37]. In addition, previous studies mostly focused on the rate of rehospitalization, not taking into account that hospitalizations and ER visits may be recurrent events in this population. This study used a matched-cohort design to compare the effect of switching from oral APs to atypical LATs (risperidone or paliperidone palmitate) with that of continuing to take oral APs on the recurrence of hospitalizations and ER visits among patients with schizophrenia who relapsed.

## Methods

### Data source

Health Insurance Portability and Accountability Act fully compliant, deidentified records were retrieved retrospectively from the Premier Perspective Comparative Hospital Database (Premier), the largest hospital-based database in the United States, covering from the first quarter of 2006 to the fourth quarter of 2010. This database provides detailed information for more than 45 million inpatient discharges and 310 million hospital outpatient visits from more than 600 acute care hospitals across all US regions. Data elements included demographics (e.g., age, gender, marital status, race, payer type), visit-level information (e.g., primary and secondary diagnoses), hospital characteristics (e.g., urban, teaching, number of beds, region), and detailed drug use information (e.g., drug name, dosage strength, dispensed quantity). As opposed to centralized health care claims recorded by insurance companies, patients’ medical information available in the Premier database comes from records collected for billing purposes at the hospital level. Institutional review board (IRB) and informed consent were not required for this study.

### Study design

**Figure 1:**
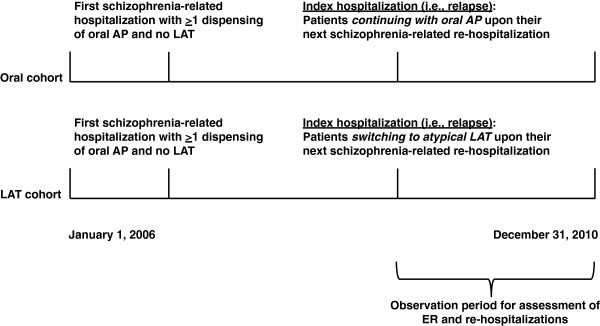
**Study design.** AP, antipsychotic; ER, emergency room; LAT, long-acting therapy.

A retrospective cohort design was used to identify patients with schizophrenia treated with APs who relapsed. More specifically, the study included adult patients (aged at least 18 years) receiving oral APs during a first schizophrenia-related hospitalization (defined as hospitalizations with (i) a primary or admitting diagnosis of schizophrenia according to International Classification of Diseases, 9th Revision, Clinical Modification [ICD-9-CM]: 295.xx; (ii) a primary or admitting diagnosis of other mental disorders [ICD-9-CM diagnosis: 290.xx-294.xx, 296.xx-319.xx] and an accompanying diagnosis of schizophrenia, or (iii) a primary or admitting diagnosis of injury and poisoning [ICD-9-CM diagnosis: 800.xx-999.xx] and an accompanying diagnosis of schizophrenia). Patients were further stratified upon the next schizophrenia rehospitalization (i.e., schizophrenia relapse) into the following mutually exclusive exposure groups: (a) patients switching to paliperidone palmitate or risperidone injectable (“atypical LAT group”) and (b) patients continuing with oral APs (“oral AP group”). The index hospitalization (i.e., schizophrenia relapse) had to occur at least 3 months before the data cutoff date (December 2010). Figure 1 depicts the study design scheme for this population.

### Study end points

End points for this study were the number of rehospitalizations and the number of ER visits occurring after the index hospitalization, which are avoidable events representing relapses not managed through outpatient services. Number of days in hospital over the entire observation period was reported. ER visits resulting in hospitalizations counted as one event for the latter end point. Additionally, the time to first rehospitalization (or ER visit) was defined as the number of months between the index hospitalization and the first rehospitalization (or ER visit) during follow-up. Finally, the frequency of events within the same month or within 1, 3, and 6 months after the index hospitalization was also reported. As depicted in Figure 1, these study end points were evaluated from the index hospitalization (i.e., relapse) to the data cutoff date (December 31, 2010). In addition, results were generated separately for all-cause visits, mental disorder–related visits (at least one diagnosis for mental disorders [ICD-9-CM diagnosis: 290.xx-319.xx]), and schizophrenia-related visits (at least one diagnosis for schizophrenia [ICD-9-CM diagnosis: 295.xx]).

### Matching algorithm

To minimize the potential of confounding factors, each patient in the atypical LAT group was matched with up to three unique patients in the oral AP group based on both propensity scores, using the 2.5 percentiles of the propensity score distribution and exact matching factors. These criteria were chosen to achieve both clinical and statistical balance between cohorts without losing a significant proportion of the atypical LAT group. Propensity scores were calculated using a multivariate logistic regression model in which being in the atypical LAT group was the dependent variable and characteristics available at the first hospitalization (type of oral AP used) and at the index hospitalization (sociodemographic characteristics, hospital characteristics, and clinical characteristics [e.g., admitting and primary diagnoses, admitting and attending physician specialty, length of ER stay, degree of severity, number of distinct AP agents used, type of oral AP agents received during the first hospitalization, suicidal behavior, surgery, discharge status, year of admission, time between first hospitalization and index hospitalization, and time between index hospitalization and data cutoff date]) were used as predictors of being in the atypical LAT group. Exact matching factors included schizophrenia as the primary diagnosis, categories of index hospitalization length of stay, psychiatry admitting physician specialty, and the number of AP agents used during the index hospitalization.

### Statistical analysis

Descriptive statistics were generated to summarize baseline characteristics and study end points. Frequency counts and percentages were used to summarize categorical variables while means and standard deviations were used for continuous variables. To adjust for the non-independence of the matched cohorts, statistical differences between cohorts were assessed using McNemar tests for categorical variables and the paired two-sided *t* tests for continuous variables.

Since a patient might experience multiple recurrences of hospitalizations and of ER visits, the Andersen-Gill extension of the Cox proportional hazard model was used to assess the impact of atypical LAT versus oral AP on the time to recurrences of these events [38]. In this extension, a subject contributes to the risk set for an event as long as the patient is under observation at the time the event occurs. Along with the hazard ratio, 95% confidence limits and *p* values were estimated. The analysis was conducted for all-cause visits and for the subset of mental disorder-related visits (at least one diagnosis of mental disorders [ICD-9-CM code: 290-319]) and of schizophrenia-related visits (at least one diagnosis for schizophrenia [ICD-9-CM diagnosis: 295.xx]). A two-sided alpha error of 0.05 was used to declare statistical significance. No adjustment was made for multiplicity. All statistical analyses were conducted using SAS® Version 9.2 (SAS Institute, Inc., Cary, NC, USA).

## Results

### Baseline characteristics

Among the 122,129 patients receiving oral APs in their first AP hospitalization, 1092 patients switched to an atypical LAT during a second schizophrenia-related hospitalization (whereas 35,841 patients continued to take oral APs). Among these patients, a total of 1032 atypical LAT patients (94.5% of all patients) were matched with 2796 oral AP patients. The baseline demographics and clinical characteristics of the matched populations at the index hospitalization are shown in Table 1. Atypical LAT and oral AP groups appeared well balanced with respect to mean age (42.1 vs 42.4 years, *p* = .5622), gender (43.6% vs 44.6% female, *p* = .5345), race, region, payer type, hospital characteristics, admitting diagnosis, admitting physician specialty, and degree of illness severity (*p* > .05 for all). The mean length of stay (16.3 vs 16.0 days, *p* = .6142), the mean time between first hospitalization and the index hospitalization (8.0 vs 7.5 months, *p* = .1186), and the mean time between index hospitalization and data cutoff date (29.8 vs 30.3 months, *p* = .2953) appeared to be not statistically significantly different between atypical LAT and oral AP groups.

**Table 1:** Baseline demographics and clinical characteristics at index hospitalization

**Variable**	**Atypical LAT**	**Oral AP**	***p *value**^**1**^
	**(*N*** **= 1032)**	**(*N*** **= 2796)**	
**Age, mean ± SD [median]**	42.1 ± 14.4 [42.5]	42.4 ± 13.7 [43.0]	.5622
**Female, *n *(%)**	450 (43.6)	1246 (44.6)	.5345
**Marital status, *n *(%)**			
Single	869 (84.2)	2364 (84.5)	.8531
Married	70 (6.8)	171 (6.1)	.3501
Other	93 (9.0)	260 (9.3)	.3217
Unknown	0 (0.0)	1 (0.0)	-
**Race, *n *(%)**			
White	483 (46.8)	1302 (46.6)	.8515
Black	327 (31.7)	908 (32.5)	.4751
Hispanic	29 (2.8)	77 (2.8)	.7488
Other	193 (18.7)	509 (18.2)	.6339
**Primary payer type, *n *(%)**			
Medicare	509 (49.3)	1429 (51.1)	.1455
Medicaid	327 (31.7)	864 (30.9)	.3670
Commercial indemnity	39 (3.8)	96 (3.4)	.6600
Self-pay	32 (3.1)	77 (2.8)	.6265
Managed care	68 (6.6)	183 (6.5)	.8717
Other	57 (5.5)	147 (5.3)	.6385
**Hospital characteristics, *n *(%)**			
Urban	920 (89.1)	2512 (89.8)	.4343
Teaching	422 (40.9)	1195 (42.7)	.1380
Large (≥500 beds)	311 (30.1)	875 (31.3)	.2576
**Region of the hospital, *n *(%)**			
South	438 (42.4)	1151 (41.2)	.5484
Midwest	247 (23.9)	703 (25.1)	.6391
West	187 (18.1)	491 (17.6)	.3662
Northeast	160 (15.5)	451 (16.1)	.2229
**Admission source, *n *(%)**			
Physician referral	294 (28.5)	761 (27.2)	.2282
ER	547 (53.0)	1530 (54.7)	.2747
Clinic referral	18 (1.7)	63 (2.3)	.1819
Court/law enforcement	26 (2.5)	56 (2.0)	.2773
Transfer from hospital	80 (7.8)	210 (7.5)	.7994
Other	67 (6.5)	176 (6.3)	.9110
**Admitting physician specialty, *n *(%)**			
Psychiatry	935 (90.6)	2579 (92.2)	.3415
Internal medicine (internist/hospitalist)	13 (1.3)	34 (1.2)	.8026
Family/General medicine	6 (0.6)	25 (0.9)	.1495
Other	76 (7.4)	158 (5.7)	.1448
Unknown	2 (0.2)	0 (0.0)	-
**Attending physician specialty, *n *(%)**			
Psychiatry	985 (95.4)	2702 (96.6)	.0366
Internal medicine (internist/hospitalist)	12 (1.2)	32 (1.1)	.9013
Family/General medicine	3 (0.3)	6 (0.2)	.4054
Other	32 (3.1)	56 (2.0)	.0258
**Most frequent admitting diagnoses, *n *(%)**			
Schizophrenia^3^	627 (60.8)	1721 (61.6)	.5098
Other mental disorders^4^	150 (14.5)	403 (14.4)	.9093
Injury and poisoning^7^	4 (0.4)	5 (0.2)	.0896
Other	21 (2.0)	61 (2.2)	.9263
Unknown	230 (22.3)	606 (21.7)	.5012
**Most frequent primary diagnoses, *n *(%)**			
Schizophrenia^3^	976 (94.6)	2686 (96.1)	.0897
Paranoid (ICD-9-CM: 295.3)	426 (43.6)	1165 (43.4)	.7467
Schizoaffective disorder (ICD-9-CM: 295.4)	370 (37.9)	1047 (39.0)	.4872
Unspecified (ICD-9-CM: 295.9)	80 (8.2)	209 (7.8)	.8782
Residual (ICD-9-CM: 295.6)	62 (6.4)	162 (6.0)	.4025
Other	38 (3.9)	103 (3.8)	.6650
Other mental disorders^4^	46 (4.5)	90 (3.2)	.2541
Diseases of the circulatory system^5^	0 (0.0)	1 (0.0)	-
Injury and poisoning^6^	8 (0.8)	12 (0.4)	.0455
Other	2 (0.2)	7 (0.3)	.7815
**Degree of severity, *n *(%)**^**7**^			
Minor	284 (27.5)	764 (27.3)	.6578
Moderate	680 (65.9)	1847 (66.1)	.9115
Major	60 (5.8)	160 (5.7)	.7687
Extreme	8 (0.8)	25 (0.9)	.3657
**Number of distinct APs used, mean ± SD [median]**^**2**^	2.2 ± 1.0 [2.0]	2.2 ± 1.0 [2.0]	1.0000
**Suicidal behavior, *n *(%)**^**8**^	115 (11.1)	303 (10.8)	.6393
**Surgery during index hospitalization, *n *(%)**	21 (2.0)	76 (2.7)	.0518
**Discharge status, *n *(%)**			
Home	853 (79.4)	2425 (80.4)	.1478
Transferred to hospice, rehabilitation center, or nursing home	64 (6.9)	230 (7.6)	.3959
Discharged/transferred to psychiatric facility	46 (4.5)	153 (5.1)	.0825
Other/Unknown	69 (6.7)	207 (6.9)	.9592
**Length of stay, days, mean ± SD [median]**	16.3 ± 19.4 [11.0]	16.0 ± 22.3 [10.0]	.6142
**Time between first hospitalization and index hospitalization, months, mean ± SD [median]**	8.0 ± 10.2 [3.0]	7.5 ± 9.8 [3.0]	.1186
**Time between index hospitalization and data cutoff date, months, mean ± SD [median]**	29.8 ± 16.1 [29.0]	30.3 ± 16.1 [29.0]	.2953
**Year of admission, *n *(%)**			
2006	200 (19.4)	550 (19.7)	.7883
2007	189 (18.3)	548 (19.6)	.1657
2008	228 (22.1)	603 (21.6)	.3887
2009	241 (23.4)	656 (23.5)	.9749
2010	174 (16.9)	439 (15.7)	.4453

AP, antipsychotics; ICD-9-CM, International Classification of Diseases, 9th Revision, Clinical Modification; LAT, long-acting therapy; SD, standard deviation.

Notes:

1. Calculated using McNemar tests for categorical variables and two-sided paired *t* tests for continuous variables.

2. Oral and injection forms of the same compound counted as one AP agent.

3. Defined as ICD-9-CM codes: 295.x.

4. Defined as ICD-9-CM codes: 290-294, 296-319.

5. Defined as ICD-9-CM codes: 390-459.

6. Defined as ICD-9-CM codes: 800-999.

7. Based on an algorithm developed by 3 M Health Information Systems (Salt Lake City, UT, USA). Constructed by considering (1) the primary admitting diagnosis, (2) the secondary diagnoses, (3) the age of the patient, and (4) the presence of certain procedures.

8. Identified using ICD-9-CM diagnosis codes for suicidal tendencies, suicidal ideation, suicide and self-inflicted injury, injury undetermined whether accidentally or purposely inflicted, and poisoning by drug, medicinal, and biological substances.

### Rehospitalizations and emergency room visits

Table 2 presents descriptive statistics on rehospitalization rates and ER visits after the index hospitalization. Over a mean 30-month follow-up period, atypical LAT patients were associated with a significantly lower mean number of all-cause rehospitalizations (1.25 vs 1.61, *p* < .0001), mental disorder-related rehospitalizations (1.24 vs 1.59, *p* < .0001), schizophrenia-related rehospitalizations (1.15 vs 1.41, *p* = .0005), and all-cause ER visits (2.33 vs 2.67, *p* = .0158) compared with oral AP patients. The frequency of all-cause rehospitalizations within the same month (0.07 vs 0.09, *p* = .0688), within 1 month (0.15 vs 0.20, *p* = .0286), within 3 months (0.30 vs 0.38, *p* = .0288), and within 6 months (0.48 vs 0.58, *p* = .0029) was consistently lower for the atypical LAT group than for the oral AP group. Similarly, frequencies for mental disorder-related rehospitalizations were significantly lower for the atypical LAT group than for the oral AP (same month: 0.07 vs 0.09, *p* = .0786; 1 month: 0.15 vs 0.20, *p* = .0360; 3 months: 0.30 vs 0.37, *p* = .0333; and 6 months: 0.48 vs 0.58, *p* = .0032). All-cause and mental disorder-related mean days in hospital were also smaller for the atypical LAT cohort (all-cause: 13.46 vs. 15.69, p = .0081; mental disorder-related: 13.44 vs. 15.62, p = .0093, schizophrenia-related: 12.79 vs. 14.28, p = .0893).

**Table 2:** Frequency of rehospitalizations and emergency room visits

	**Atypical LAT**	**Oral AP**	***p *value**^**1**^
	**(*N*** **= 1032)**	**(*N*** **= 2796)**	
**Rehospitalizations**			
***All-cause rehospitalizations***			
Number of rehospitalizations, mean ± SD	1.25 ± 2.09	1.61 ± 2.82	<.0001
Mean number of days in hospital, mean ± SD	13.46 ± 27.48	15.69 ± 30.49	.0081
Time to first rehospitalization, months, mean ± SD	7.47 ± 9.30	7.04 ± 8.86	.2362
Frequency of rehospitalizations, mean ± SD			
Within the same month	0.07 ± 0.27	0.09 ± 0.31	.0688
By 1 month	0.15 ± 0.41	0.20 ± 0.49	.0286
By 3 months	0.30 ± 0.62	0.38 ± 0.76	.0288
By 6 months	0.48 ± 0.84	0.58 ± 1.05	.0029
***Mental disorder–related rehospitalizations***^***2***^			
Number of rehospitalizations, mean ± SD	1.24 ± 2.08	1.59 ± 2.79	<.0001
Mean number of days in hospital, mean ± SD	13.44 ± 27.46	15.62 ± 30.41	.0093
Time to first rehospitalization, months, mean ± SD	7.46 ± 9.31	7.02 ± 8.84	.2451
Frequency of rehospitalizations, mean ± SD			
Within the same month	0.07 ± 0.27	0.09 ± 0.31	.0786
By 1 month	0.15 ± 0.41	0.20 ± 0.49	.0360
By 3 months	0.30 ± 0.62	0.37 ± 0.76	.0333
By 6 months	0.48 ± 0.84	0.58 ± 1.05	.0032
***Schizophrenia–related rehospitalizations***^***3***^			
Number of rehospitalizations, mean ± SD	1.15 ± 2.00	1.41 ± 2.54	.0005
Mean number of days in hospital, mean ± SD	12.79 ± 27.07	14.28 ± 29.14	.0893
Time to first rehospitalization, months, mean ± SD	7.39 ± 9.32	7.16 ± 8.99	.4063
Frequency of rehospitalizations, mean ± SD			
Within the same month	0.06 ± 0.25	0.08 ± 0.29	.0854
By 1 month	0.14 ± 0.39	0.18 ± 0.46	.1834
By 3 months	0.28 ± 0.60	0.34 ± 0.72	.2080
By 6 months	0.45 ± 0.81	0.52 ± 0.97	.0947
**ER Visits**			
***All-cause ER visits***			
Number of ER visits, mean ± SD	2.33 ± 5.58	2.67 ± 6.38	.0158
Time to first ER visit, months, mean ± SD	6.91 ± 8.82	6.79 ± 8.84	.6060
Frequency of ER visits, mean ± SD			
Within the same month	0.08 ± 0.31	0.09 ± 0.34	.4026
By 1 month	0.21 ± 0.60	0.25 ± 0.66	.0482
By 3 months	0.44 ± 1.18	0.50 ± 1.15	.1375
By 6 months	0.75 ± 1.84	0.78 ± 1.61	.3822
***Mental disorder–related ER visits***^***2***^			
Number of ER visits, mean ± SD	1.95 ± 4.73	2.08 ± 4.33	.0881
Time to first ER visit, months, mean ± SD	7.41 ± 9.27	7.34 ± 9.41	.6870
Frequency of ER visits, mean ± SD			
Within the same month	0.07 ± 0.28	0.08 ± 0.30	.5467
By 1 month	0.18 ± 0.54	0.21 ± 0.55	.2015
By 3 months	0.38 ± 1.03	0.41 ± 0.94	.4573
By 6 months	0.64 ± 1.46	0.64 ± 1.29	.6932
***Schizophrenia–related ER visits***^***3***^			
Number of ER visits, mean ± SD	1.50 ± 3.36	1.54 ± 3.22	.2637
Time to first ER visit, months, mean ± SD	7.77 ± 9.57	7.73 ± 9.56	.7920
Frequency of ER visits, mean ± SD			
Within the same month	0.06 ± 0.26	0.06 ± 0.27	.5023
By 1 month	0.15 ± 0.47	0.16 ± 0.45	.5133
By 3 months	0.31 ± 0.90	0.32 ± 0.77	.8963
By 6 months	0.50 ± 1.21	0.50 ± 1.04	.8894

AP, antipsychotics; ER, emergency room; ICD-9-CM, International Classification of Diseases, 9th Revision, Clinical Modification; LAT, long-acting therapy; SD, standard deviation.

Notes:

1. Calculated using two-sided paired *t* tests.

2. At least one diagnosis of mental disorders (ICD-9-CM codes: 290-319) during rehospitalization or ER visit.

3. At least one diagnosis of schizophrenia (ICD-9-CM codes: 295) during rehospitalization or ER visit.

### Recurrence of events

**Figure 2:**
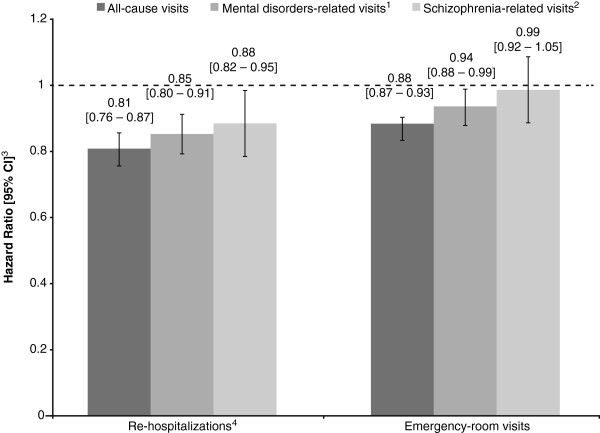
**Risk of rehospitalizations and ER visits using Cox proportional hazard models with recurrent events (Andersen-Gill method): atypical LATs relative to oral APs.** AP, antipsychotic; ER, emergency room; LAT, long-acting therapy. Notes: 1. At least one diagnosis of mental disorders (ICD-9 codes: 290-319) during rehospitalization or ER visit. 2. At least one diagnosis of schizophrenia (ICD-9 codes: 295) during rehospitalization or ER visit. 3. The vertical bars represent the 95% CIs. A HR <1 indicates that patients receiving atypical LATs were associated with lower rehospitalization and ER visit rates than patients treated with oral APs. 4. ER visits resulting in rehospitalizations were counted as one event.

Figure 2 presents the results of the Cox proportional hazards model with recurrent events using the Andersen-Gill extension. The risk of all-cause rehospitalizations was significantly lower for the atypical LAT group than for the oral AP group (hazard ratio [HR] 0.81, 95% confidence interval [CI] 0.76–0.87, *p* < .0001). Consistently significant results were found for all-cause ER visits (HR 0.88, 95% CI 0.87–0.93, *p* < .0001).

Similarly, significantly lower risks for mental disorder-related events were observed for atypical LAT patients relative to oral AP patients (rehospitalizations: HR 0.85, 95% CI 0.80–0.91, *p* < .0001; ER visits: HR 0.94, 95% CI 0.88–0.99, *p* = .0285). A lower risk of schizophrenia-related rehospitalizations was found for the atypical LAT group (HR: 0.88, 95% CI 0.82-0.95, *p* = .0007), whereas no difference between cohorts was found for schizophrenia-related emergency-room visits (HR: 0.99, 95% CI 0.92-1.05, *p* = .6851).

## Discussion

This real-world retrospective study compared the recurrence of hospitalizations and ER visits between relapsed patients with schizophrenia treated with atypical LATs and those treated with oral AP agents. The results showed a 19% to 12% significantly lower likelihood of all-cause rehospitalizations and ER visits for the atypical LAT group relative to the oral AP group. Results were similar when restricting the analysis to the subset of events associated with a diagnosis for mental disorders.

In the absence of direct clinical assessment data, this study used rehospitalizations and ER visits as a proxy for instances of relapses. This is warranted as hospitalizations are related to a wide range of schizophrenic outcomes such as suicidal attempt, violence, and medication side effects [9,27,39-41]. Schizophrenia disease is often characterized by episodes of relapse alternating with periods of complete or partial remission [8]. Successive relapses can reduce the degree and duration of the following remission, worsen disability, and increase refractoriness to future treatment [8,42]. Relapses are associated with high medical and non-medical costs as well as productivity loss [40]. Thus, rehospitalization, which is frequently the most expensive healthcare cost component for psychotic patients, is a relevant relapse measure [40].

Rehospitalizations and ER visits were analyzed in a relapsed population. Patients receiving atypical LAT instead of oral AP may be more difficult to treat, with poorer adherence to medication and/or with more severe symptoms. In the absence of patients’ complete medical history, imposing a previous hospitalization with AP utilization prior to the index hospitalization served as an exact matching criterion to identify similar groups of patients. Along with the choice of the study design, the matching algorithm used in the current study contributed to diminishing the risk of selection bias resulting from confounding factors between groups. A comprehensive list of demographics and clinical characteristics available during the index hospitalization was included in the exact matching algorithm and propensity scores model. Moreover, the sample sizes allowed the authors to match each atypical LAT patient with up to three oral AP patients, thus increasing the statistical power of the analyses.

The descriptive results of this study showed that atypical LAT patients had a significantly lower rate of rehospitalizations compared with oral AP patients in addition to a non-significantly longer time period between the index hospitalization and the first rehospitalization (Table 2). These two factors were combined in one statistical analysis, the Andersen-Gill extension of the Cox regression model, to calculate the risk of time to multiple recurrences of hospitalizations and ER visits. The Andersen-Gill model uses a counting process approach which relates the intensity function of event recurrences to the covariates multiplicatively where all events contribute equally to the hazard function. The hazard ratios of recurrent events calculated using this method confirmed the descriptive results that patients using atypical LATs have a lower risk of rehospitalizations and ER visits compared with oral AP patients.

This study corroborates the findings from other studies that have found a beneficial effect of atypical LATs in terms of rehospitalizations [9,20,22,27-37]. Some of these studies used a pre-post study design, where each patient acted as his or her own control, and found that LATs were associated with a decrease in hospitalizations ranging from 34% to 89% [20,22,27-34]. The current analysis based on a matched cohort design found that LATs were associated with a 19% reduction in the risk of recurrence of hospitalizations when compared to matched oral AP patients. The slightly smaller effect found in the current analysis may be explained in part by the study design, where the comparison was made on relapsed oral AP patients, which are likely an healthier population than the subset of patients switching to LATs (and hence are expected to have fewer rehospitalizations). Focusing on the relapsed population (patients already experiencing a second schizophrenia-related hospitalization) and the matching approach have helped to address this bias, but it is possible that unobservable characteristics were still different between cohorts, therefore explaining the smaller effect found here. However, the general consistency of the results throughout different methods, study designs, and study populations suggests that atypical LATs may be more effective than oral APs in avoiding patient relapse. Moreover, it has been shown that inpatient care can account for up to two-thirds of the total direct healthcare costs for schizophrenia patients in the U.S. [3,4,6]. Thus, the 19% decrease in the risk of recurrence of rehospitalization found in the current study suggest that the increased use of LATs compared to oral AP may be associated with substantial cost savings in relapsed patients with schizophrenia. Further studies analyzing costs of hospitalizations between patients using atypical LATs and matched oral AP patients are warranted.

The present study has several limitations: (a) The data were subject to billing inaccuracies and missing data. (b) As the Premier network regroups only a subset of facilities in the United States, the history of rehospitalizations and ER visits for a given patient may be incomplete. Moreover, it was not possible in the Premier database to link patient records across facilities, and, therefore, to know whether or not the entire continuum of care of patients was captured. (c) Patients were matched based only on information available during hospitalizations occurring at the same hospital as no information on services received outside the hospital or on pharmacy utilization was available, potentially limiting the ability to fully adjust for differences in baseline severity of the disease. Additionally, rehospitalizations recorded in the database were only those occurring at the same hospital. Therefore, the rates of hospitalization may be underestimated; however, it can be assumed that the rates were underreported equivalently between cohorts. (d) The lack of clinical data (e.g., PANSS score) prevented us from assessing patients’ disease severity and/or the occurrence of a clinically validated relapse event. Although all these limitations exist, they should not have introduced bias in one cohort versus the other because neither impacts the relative differences found between cohorts.

## Conclusions

This large hospital database analysis demonstrated that relapsed patients with schizophrenia treated with atypical LATs were associated with a 19% lower likelihood of rehospitalizations compared with patients receiving oral APs. Atypical LATs were also associated with significantly lower ER visit rates and mental disorder-related and schizophrenia-related rehospitalization rates compared with oral APs. These findings suggest that the efficacy and patient tolerability benefits associated with improved patient adherence to the injectable form of the second-generation antipsychotics reduce the demand for hospitalizations and ER visits among relapsed patients with schizophrenia. In addition to the clinical benefits, atypical LATs offer promise for cost savings because of reduced inpatient hospital utilization.

## Competing interests

M-H Lafeuille, F Laliberté-Auger, and P Lefebvre are employees of Groupe d’analyse, Ltée.

C Frois and MS Duh are employees of Analysis Group, Inc.

J Fastenau is an employee of Janssen Scientific Affairs, LLC, and a Johnson & Johnson stockholder.

## Authors’ contributions

MHL, FL-A, PL, CF, MSD, JF were responsible for the design, data collection, and writing. MHL, FL-A, PL, and MSD were responsible for the statistical analyses. All authors read and approved the final manuscript.

## Pre-publication history

The pre-publication history for this paper can be accessed here:

http://www.biomedcentral.com/1471-244X/13/221/prepub
